# Ganglions

**Published:** 1891-01-10

**Authors:** 


### GANGLIONS.

This absurd name ought to be discarded in favour of Hygroma, applied to these cysts by German surgeons when they do not use the vernacular *Schleim-bentel,*" slime-bag," *i.e.*, mucous cyst. Though sometimes connected with or prolongations of the synovial sacs of the carpus, they are often quite independent, attached to the tendons or fascia, or entirely free. In the first case evacuation and pressure may effect a cure, but when they do not communicate with a joint they should certainly be excised like other cystic tumours. Dr. F. Roeder has discussed the operative treatment of a number of various kinds, but one of the most remarkable is reported by Von Büngner. The patient was an old man aged 74 who 37 years ago had undergone amputation of the thigh in the upper third in consequence of a gun-shot injury with compound and comminuted fracture of the bone. Latterly an enormous tumour had developed behind the great trochanter in the stump, projecting both in front and behind, to the size of an infant's head. From it prolongations extended anteriorly inwards in a horizontal direction, and posteriorly upwards. When evacuated 1080 ccms. of sero-sanguinolent fluid, were drawn off, with a number of fibrinous clots, their combined bulk amounting to 127 cubic centimetres. The sac when excised was found to have three coats, the outermost a sort of fibrous sheath, and the innermost formed of soft fibrinous material; the clots in the sac appeared to be derived from the latter, which was in process of disintegration, rather than to have arisen from coagulation of the fibrin in the fluid. The wound healed by first intention.

